# Potential Misdiagnosis between COVID-19 and Dengue Infection Using Rapid Serological Test

**DOI:** 10.3390/idr13020050

**Published:** 2021-06-07

**Authors:** Siti Qamariyah Khairunisa, Ilham Harlan Amarullah, Siti Churrotin, Anisa Lailatul Fitria, Mochammad Amin, Maria Inge Lusida, Soegeng Soegijanto

**Affiliations:** 1 Institute of Tropical Disease, Universitas Airlangga, Surabaya 60115, Indonesia; amarullah@staf.unair.ac.id (I.H.A.); siti_churrotin@staf.unair.ac.id (S.C.); anisalailatulfitria@gmail.com (A.L.F.); amintdc@gmail.com (M.A.); ingelusida@itd.unair.ac.id (M.I.L.); tu.soerya_rsab@yahoo.co.id (S.S.); 2 Department of Microbiology, Faculty of Medicine, Universitas Airlangga, Surabaya 60131, Indonesia; 3 Department of Pediatric, Faculty of Medicine, Universitas Airlangga, Surabaya 60131, Indonesia

**Keywords:** cross-reactivity, COVID-19, Sars-Cov 2, dengue, rapid serological test

## Abstract

The coronavirus disease 2019 (COVID-19) pandemic that has a significant rapid transmission is an international public health concern. Several dengue-endemic countries reported similar clinical and laboratory features between COVID-19 and dengue in the early incubation period, and thus discerning the infection is difficult. As a dengue-endemic country, Indonesia also poses the same challenge during the COVID-19 outbreak. This current study analyzed the IgG and IgM profiles from COVID-19 patients by using a serological SARS-CoV-2 and dengue rapid test. In addition, 38 sera from healthy individuals (pre-COVID-19 date) were analyzed using a dengue rapid test. Among 120 samples, 4 samples indicated dengue IgG positive. However, IgM, NS1, and RT-PCR analyses showed negative results. Interestingly, regarding seropositivity of NS1 and DENV IgG from healthy individuals (pre COVID-19 infection), two samples were positive DENV IgG, while one of them was positive NS1. This suggested that in the dengue-endemic area, many people have already experienced dengue and have immunity against dengue virus. There is also the possibility of antibody cross-reactivity between COVID-19 and dengue infection. This also emphasizes the high demand for a rapid method with high sensitivity and specificity that can distinguish between SARS-CoV-2 and dengue.

## 1. Introduction

Human Severe Acute Respiratory Syndrome Coronavirus 2 (Sars-Cov-2) was discovered for the first time in November 2019 in Wuhan, China. As of March 2020, this virus has spread worldwide with a total of 115,289,961 infection cases and 2,564,560 deaths [1]. This virus causes a pulmonary health problem, namely, coronavirus disease 2019 (COVID-19). The first COVID-19 case in Indonesia was reported in early March 2020. Since then, the virus has spread rapidly. Within one year, the Indonesian government reported 1,353,834 confirmed positive cases with 36,271 deaths [2].

The emergence of COVID-19 has become a serious challenge for government and public health workers in dengue-endemic countries. Some countries in South East Asia regions have reported covert COVID-19 as dengue infection in the early phase of the incubation stage. Yan g et al. also mentioned that there were similarities in clinical and laboratory features between dengue infection and COVID-19 for two infected patients in Singapore [3]. Initially, the two patients were clinically diagnosed and treated with dengue infection. This was also supported by the positive result of the dengue serological test. However, their symptoms became more severe, and a further analysis confirmed them COVID-19 positive [3]. Moreover, Thailand has registered the first death of a COVID-19 patient that was previously misdiagnosed for dengue virus infection, as skin rashes were present and this commonly indicates dengue infection symptoms [4]. The evidence that COVID-19 was able to camouflage in the early phase of the infection stage and the long period of emerging severity allows for the possibility of misdiagnosis [5].

As a dengue-endemic country, Indonesia also poses the same challenge during the COVID-19 outbreak. Three cases of suspected COVID-19 dengue co-infection were also reported at a Bali hospital where the patients had a positive infection result based on the dengue serological test which included dengue virus (DENV) NS1 antigen and anti-dengue IgM/IgG. Their condition was getting worse on the second week, and a further investigation revealed that also reactive to the COVID-19 antibody test. This study then suggested co-infection between dengue and COVID-19 [6]. A coinfection case between arbovirus and coronavirus in Indonesia was reported in 2015–2016. A study found co-infection between type 3 dengue virus and enterovirus d68 for middle east respiratory syndrome coronavirus (MERS-COV) suspected patient in Indonesia [7]. From January to November 2020, 95,893 dengue infections with 661 deaths were reported in Indonesia [8]. Thus, potential co-infection of COVID-19 and dengue virus, as well as antibody cross-reactivity, might impact clinical manifestation and diagnosis.

COVID-19 has a broad spectrum of clinical manifestations ranging from asymptomatic, mild, moderate, to severe levels. The most common symptoms are fever, dry cough, and tiredness. An infected individual might also develop less common symptoms such as aches, pains, sore throat, diarrhea, nasal congestion, headache, complete loss of smell (anosmia), skin rashes, and white fingers. Severe symptoms patients face are troubled breathing, constant pain or pressure on the chest, loss of speech, confusion, stroke, and blue lips or face. When the patient’s condition gets worse, sepsis and septic shock might appear to indicate a critical status. Some of the mild and moderate symptoms due to COVID-19 infection such as fever, and skin rashes are similar to dengue infection [9]. In addition, a previous study reported that approximately 80% of COVID-19 patients had mild to moderate severity levels with unspecific manifestations [10]. Therefore, it is a new challenge for health care institutions to cope with COVID-19 in dengue-endemic regions.

The information on co-infection and cross-reactivity between dengue and COVID-19 is still limited. Therefore, epidemiological research regarding COVID-19 in dengue-endemic areas is urgently required. This current study analyzed the IgG and IgM antibodies profiles in the plasma from COVID-19 patients in Surabaya to identify possibilities of co-infection and cross-reactivity. Moreover, the research was conducted in Surabaya since the city became a red zone, meaning it has the highest number of confirmed positive COVID-19 cases in the East Java province. This study is expected to provide useful information and contribute to a better patient management system concerning COVID-19 in dengue-endemic regions.

## 2. Materials and Methods

### 2.1. Ethics Statement

This study was conducted after obtaining approvals from the Ethics Committees of Universitas Airlangga (123/KEP/2021). All study participants were enrolled in the research after providing written informed consent forms.

### 2.2. Sample Collection from COVID-19 Patients

A total of 123 anonymous plasma were obtained from COVID-19 patients who were tested for COVID-19 in the Institute of Tropical Disease in Surabaya. All of the patients were confirmed positive by real-time PCR on nasopharyngeal swab samples by the standard assay targeting envelope (E), N, and RdRp genes based on the World Health Organization. Blood was collected within one week after positive case confirmation. After that, the plasma was separated from the blood and stored at −80 °C. In addition, we tested 38 sera from healthy individuals from 2014 (pre-COVID-19 date) which have been kept in our laboratories. Written informed consent forms were agreed upon and signed by each individual before sample collection. Metadata consisting of clinical symptoms and patient identities were confirmed through medical records.

### 2.3. Serological Test

All of the plasmas were analyzed using three rapid test kits. SARS-CoV-2 antibodies were examined using anti-Sars-Cov-2 IgM/IgG (Wuhan Unscience Biotechnology, Wuhan, China) and anti-Sars-Cov-2 IgM/IgG (Vazyme Medical Technology, Nanjing, China). Dengue antibodies were determined using DENV IgM/IgG (SD Biosensor, Suwon-si, Korea). Samples showing positive dengue antibodies from COVID-19 patients were confirmed using NS1 DENV (SD Biosensor, Suwon-si, Korea). In addition, sera from healthy individuals were analyzed using dengue rapid diagnostic test (RDT). All serological tests were performed according to the manufactured instruction kits.

### 2.4. Molecular Test

The presence of viral RNA DENV from samples with positive dengue antibodies from COVID-19 patients was confirmed by RT-PCR (Sansure Biotech Inc, Changsha, China). The RNA extraction was done using the QIAamp Viral Mini Spin Kit (Qiagen, Hilden, Germany). Briefly, the viral RNA was reversely transcribed to cDNA by using the SuperScript III First-Stand Synthesis Kit (Invitrogen, Carlsbad, CA, USA) with the reverse primer, D2 (5′-TTGCACCAACAGTCAATGTCTTCAGGTTC-3′). DENV-1, DENV-2, DENV-3, and DENV-4 genomes were amplified by a multiplex PCR using GoTaq Green master mix (Promega, Madison, WI, USA), along with the sense primer D1 (5′-TCAATATGCTGAAACGCGCGAGAAACCG-3′) and the serotype-specific reverse primers (TS1: 5′-CGTCTCAGTGATCCGGGGG-3′; TS2: 5′-CGCCACAAGGGCCATGAACAG-3′; TS3: 5′-TAACATCATCATGAGACAGAGC-3′; TS4: 5′-CTCTGTTGTCTTAAACAAGAGA-3′) to generate 482, 118, 290, and 392-bp fragments, respectively. The DENV serotype was determined by the size of the amplified fragments [11].

## 3. Results

### 3.1. Serological SARS-CoV-2 Test

The presence of antibodies was examined using two rapid detection kits. Among the samples, there were three plasmas which were not successfully analyzed due to technical difficulties. Thus, only 120 samples were used to conduct analysis and draw a conclusion. Comparison of the antibodies detections from both kits showed similarities and discrepancies in 94/120 (78.33%) and 26/120 (21.67%) samples, respectively.

**Figure 1:**
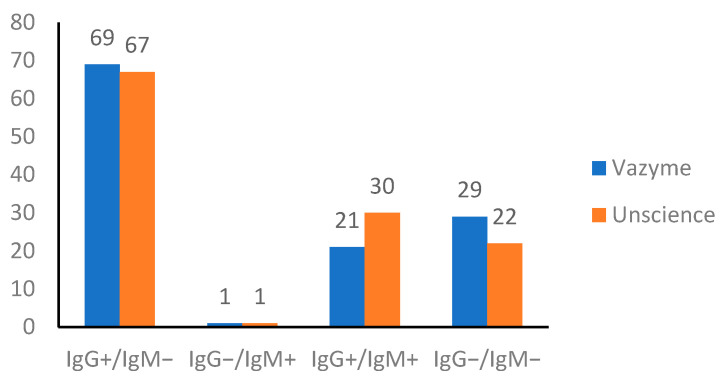
Results of comparison between Vazyme and Unscience.

The comparison of IgG and IgM detections was provided in Figure 1. Despite the fact that the UNscience detected slightly higher antibody levels in the samples, both kits provided comparable results.

### 3.2. Serological Dengue and NS1 Tests

All of the plasmas of COVID-19 patients were also subjected to IgG and IgM antibodies test of DENV. Four samples indicated positive results (Table 1).

**Figure 2:**
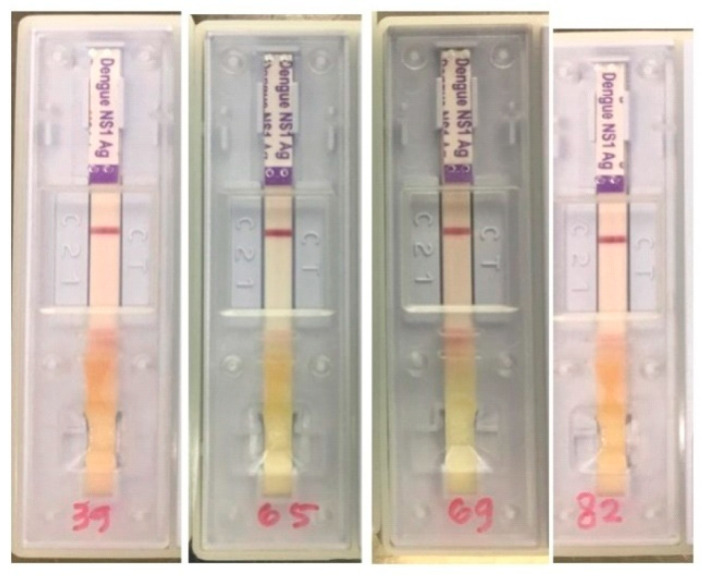
NS1 serological test of positive dengue antibodies samples.

The samples only showed DENV IgG positive result albeit faint appearance since the bands’ intensity, indicating the presence of dengue IgG, was not high. Nevertheless, it was still counted as a positive result. Another limitation of the dengue rapid kit employed (SD Biosensor, Suwon-si, Korea) is that the limit of detection (LOD) is not mentioned by the manufacturer. However, according to the kit manual, the specificity and sensitivity of IgG, IgM, and NS1 detection show a high correlation compared to RT-PCR and ELISA (≥92.9%). Interestingly, among the samples, only one sample (no. 69) was negative SARS-CoV-2 IgG and IgM. Subsequently, four samples were also investigated using a rapid NS1 test. The results showed none of the samples indicated detectable NS1 (Figure 2).

As many as 38 sera from healthy individuals in Surabaya were collected using the dengue RDT (pre-COVID-19 date). According to Table 2, one of the samples showed positive NS1 and positive DENV IgG. The results indicated that the individual could be in the early phase of dengue infection. The other sample showed positive DENV IgG but negative NS1. This result indicated the individual had the dengue antibody as a result of the previous infection.

### 3.3. Amplification of DENV Serotype

**Figure 3:**
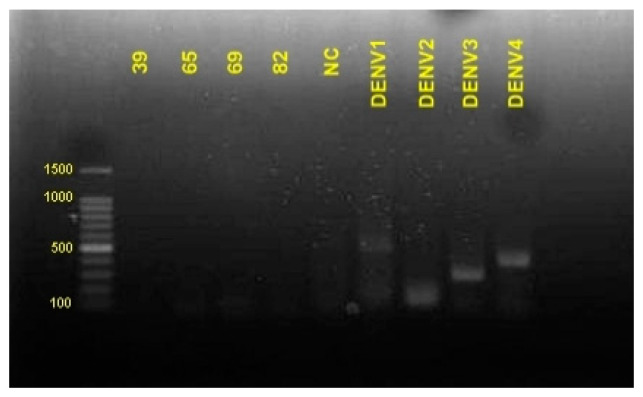
NS1 serological test of positive dengue antibodies samples.

A total of 4 of 120 samples from COVID-19 patients showing positive dengue antibodies were also confirmed for the presence of viral RNA of DENV by RT-PCR. The results showed the samples were negative DENV1, DENV2, DENV3, and DENV4 (Figure 3).

## 4. Discussion

The rapid serological test using plasmas from confirmed COVID-19 patients were analyzed. The results of two COVID-19 serological kits were not identical but still relatively comparable. This might be due to the discrepancy in sensitivity and specificity of both methods. Furthermore, the difference in sample compatibility and method principles could also possibly affect the outcomes. The Vazyme is suitable for serum and plasma samples [12], while the UNscience can also be used for the whole blood samples [13]. The Vazyme has sensitivity of 91.54% (95% CI: 86.87%, 94.65%) and specificity of 97.02% (95% CI: 94.74%, 98.33%) according to the kit manual, while the UNscience has a clinical sensitivity of 98.511% (95% CI: 96.788%, 99.452%) and specificity of 88.208% (95% CI: 83.086%, 92.221%) according to the manual.

Although all of the samples were taken from patients with COVID-19, not all of them showed a positive result of SARS-CoV-2 antibodies. The number of samples with a seronegative result based on the Vazyme and UNscience were 29 and 22, respectively. To date, the response of the host immune system and variation of antibodies profiles towards COVID-19 are still not fully understood. Table 3 shows the results of PCR test and its correlation with the serological test. Patients’ age and comorbidities might play a role in the development of SARS-CoV-2 antibodies. A study in China revealed that seroconversion of SARS-CoV-2 antibodies varied among patients. The IgG and IgM could emerge simultaneously or sequentially. Seroconversion occurred within 20 days after symptoms onset with day 13 as the median. Interestingly, two patients displayed negative IgG and IgM through the hospitalization period [14]. Another study in London, UK, also demonstrated that 2.0–8.5% of patients with severe COVID-19 did not show IgG 3–6 weeks after infection. It suggested that mild infections and younger age had a low probability of seroconversion. On the contrary, factors such as older age, non-white race, and hypertension might contribute to the seroconversion [15].

A total of 4 out of 120 samples were serologically positive for dengue IgG while the NS1 test and RT-PCR test showed a negative result. There are two possibilities that could explain the results. Firstly, these four patients had had dengue infection before the hospital admission due to COVID-19. Therefore, the antibody remained circulating in the blood although the virus had already gone. Dengue antibody response in the post infection can last for a long time. IgM circulates in the body up to 2 to 6 months, while IgG persists longer, generally up to 6 months to 2 years after dengue primary infection. Furthermore, upon secondary infection, IgG reacts earlier with higher levels and a longer deployment period [16].

Thus, the presence of higher dengue IgG compared to IgM in healthy residents of dengue-endemic area is plausible. One study showed that of 910 healthy adult donors in Saudi Arabia, 38.9% were seropositive to IgG, while positive IgM and NS1 were found in 5.5% and 5.3%, respectively [17].

Another seroprevalance screening in Guangzhou observed among 2085 serum samples, IgG and IgM positive rates were 11.80% and 3.98%, respectively [18]. To the best of the researcher’s knowledge, there is no seroprevalance study yet to assess the positive rate of healthy asymptomatic people in Indonesia. Therefore, we conducted additional RDT analysis to healthy samples from 2014 (pre-COVID-19 date) which have been kept in our laboratories. Due to the limitations of samples and materials, only 38 samples were included. The results indicated two samples were reactive to IgG detection, of which showed positive NS1. Based on the data, this study suggested the rate of dengue IgG among healthy people in Indonesia was 5.26%. However, to get more reliable data, this study suggests performing a similar experiment with a higher number of samples and repeating testing in future studies.

The second possible reason is the presence of antibody cross-reactivity between COVID-19 and dengue infection in the samples. This condition could become a significant hurdle, especially in the dengue-endemic areas since it could give false-positive results and misdiagnoses, there by delaying appropriate patients treatment. Since the first report of COVID-19 and dengue cross-reactivity in Singapore, several similar cases have been reported in other countries such as Thailand [19], Indonesia [6,20], and Italy [21].

A similar study was done by Marsha et al. using the same SD Biosensor to assess the antibodies of 33 positive COVID-19 samples from asymptomatic patients. Two samples were dengue IgG positive, and 4 samples of 19 samples with positive COVID-19 IgM were also positive for dengue IgG [22]. The SD Biosensor kit has high sensitivity and specificity, thus the diagnostic errors probability is low. According to the kit manual, the performance of the SD biosensor had been tested to determine the sensitivity and specificity in detecting dengue. The IgM detection compared with ELISA indicated 97.5% and 96.6% sensitivity and specificity, respectively. Likewise, IgG was also comparable to ELISA, showing a sensitivity of 97.2% and specificity of 96.2%. NS1 detection was compared to RT-PCR giving sensitivity and specificity of 92.9% and 98.7%, respectively. A study on the sensitivity and specificity from different dengue antibody RDT has been done by Kok-Siang Yow et al. The results indicated that Standard Q (SD Biosensor) had the highest sensitivity in the detection of IgM and NS1 compared to Multisure, Bioline, and careers. All RDTs had high specificity for dengue NS1 detection (100%). The IgM detection was also high at 100% except for Multisure (96.7%) [23].

The cross-reactivity was not only observed from COVID-19 patients with dengue serology and vice versa. A study on the serological analysis of dengue samples before September 2019 (pre-COVID-19) found 21 out of 95 samples were false positives for COVID-19 rapid tests [24]. A similar study in India observed 5 samples of 13 confirmed dengue serum collected in 2017 were false positives for COVID-19 [25]. An in silico study found that there was a high similarity between spike proteins of SARS-CoV-2 and dengue’s envelope proteins [24]. This level of similarity was also observed from envelope proteins of Zika virus. On the contrary, the comparison to envelope proteins of West Nile virus (WNV) displayed a low similarity in structure. Such a similarity could elaborate the reasons for antibody cross-reactivity between SARS-CoV-2 and the dengue virus.

Among the samples included, only one sample showed positive dengue antibodies, while the serological test of COVID-19 was negative. This might potentially indicate COVID-19 and dengue co-infection. Surabaya, where the samples were collected, was often monitored for dengue virus existence. Four dengue serotypes (DENV1–4) were found in humans and mosquitoes in the past two decades despite the difference in the time and dominance level [11,26,27]. Moreover, the occurrence of COVID-19 and dengue infection was also observed and reported in several dengue-endemic countries such as Brazil [28,29], Thailand [30], and Reunion Island [31].

Despite the useful results, there are some limitations to this current study. First, the samples were collected from the patients one time. It would be better to take samples at several time point intervals. Thus, whether the seronegative cases were true or in the undetectable low-level antibodies stage could not be elaborated. Second, the viral load was not examined, while the antibody level could correlate with the amount of virus in the body. Third, unfortunately, this study could not perform a neutralization assay due to the unavailability of the materials. Therefore, future studies should document the viral load, serologic responses in the body, and perform a neutralization assay to samples. In addition to these recommendations, prospective studies should consider testing serological SARS-CoV-2 on positive dengue plasma samples collected before the COVID-19 pandemic to gain more insights into cross-reactivity.

## 5. Conclusions

In conclusion, using the serological RDT to determine COVID-19 or dengue infection might lead to misdiagnosis, while the gold standard of COVID-19 detection is Real-Time PCR. However, the usage of serological RDT for dengue infection is popular due to its simplicity and affordability particularly in developing countries. Thus, IgG/IgM RDT cannot be used by itself. We recommend prioritizing the NS1 result for detection of dengue infection during the COVID-19 pandemic. Ideally, patients are examined for both diseases using the PCR-based method. Overall, the findings alert the health care in dengue-endemic regions to improve awareness and accurate diagnoses. The potential of concomitant infection should also be considered to prevent dangerous impacts on patients. This also emphasizes that a rapid serological method with high sensitivity and specificity is required to distinguish between SARS-CoV-2 and dengue infections.

## Figures and Tables

**Table 1:** Samples from COVID-19 patients with positive dengue antibodies.

Sample No.	COVID-19 *				Dengue		
	Vazyme		Unscience		IgG	IgM	NS1
	IgG	IgM	IgG	IgM			
39 ****	** +	*** −	+	−	+	−	−
65 ****	+	+	+	+	+	−	−
69 ****	−	−	−	−	+	−	−
82 ****	+	−	+	+	+	−	−

* nasopharyngeal swab samples were confirmed COVID-19 positive by a real-time PCR. ** + sign denotes positive results. *** − sign denotes negative results. **** ID sample.

**Table 2:** The characteristics of healthy individuals (pre COVID-19 date) tested by dengue RDT (*n* = 38) in Surabaya, Indonesia.

Sample	IgG (+), IgM (+)	IgG (+), IgM (−)	IgG (−), IgM (+)	IgG (−), IgM (−)
NS1 (+) *	0/38	1/38	0/38	0/38
NS (−) **	0/38	1/38	0/38	0/38

* sample indicated dengue NS1 positive. ** sample indicated dengue NS1 negative.

**Table 3:** The data of PCR test and its correlation with serological test.

Sample Number	Blood Collection (Days after Positive COVID-19 Confirmation)	qPCR			Serological Detection			
		CT Value			Vazyme		UNscience	
		Orf1ab	N	Host	IgG	IgM	IgG	IgM
1	0	37.46	39.86	23.45	+	−	+	+
2	0	NA *	NA	NA	+	−	+	−
3	0	NA	NA	NA	+	−	+	−
4	3	NA	NA	NA	+	+	+	+
5	3	NA	NA	NA	+	−	+	+
6	3	36.77	37.78	− **	+	−	+	−
7	3	NA	NA	NA	+	−	+	−
8	8	NA	NA	NA	−	−	−	−
9	9	37.1	37.59	29.92	−	−	+	−
10	10	NA	NA	NA	+	−	+	−
11	9	NA	NA	NA	−	−	+	+
12	9	NA	NA	NA	+	−	+	−
13	4	37.66	39.05	−	+	−	+	−
14	9	37.3	37.5	−	+	−	+	+
15	3	33.04	33.59	24.07	+	+	+	+
16	1	22.96	24.5	−	−	−	−	−
17	9	32.51	33.17	20.44	+	−	+	+
18	8	36.15	35.92	24.89	+	+	+	+
19	9	36.29	−	36.87	−	−	−	−
20	9	36.31	38.5	24.6	+	−	+	+
21	11	NA	NA	NA	+	−	+	+
22	7	27.42	27.7	20.51	+	−	+	−
23	0	33.67	33.96	20.85	+	−	+	−
24	2	38.38	39.67	24.59	+	−	+	+
25	3	33.95	34.38	23.86	+	−	+	−
26	3	37.69	37.26	22.27	+	−	+	+
27	6	36.35	38.53	23.1	+	+	+	+
28	1	37.4	38.8	21.58	+	−	+	−
29	1	30.29	30.53	20.72	+	−	+	−
30	4	NA	NA	NA	−	−	−	−
31	8	NA	NA	NA	−	−	−	−
32	4	28.83	29.71	22.04	−	−	−	−
33	4	NA	NA	NA	−	−	−	−
34	5	35.86	36.56	24.49	+	−	+	−
35	5	38.83	22.28	−	−	−	−	−
36	5	36.14	38.28	24.11	−	−	−	−
37	1	35.58	37.96	23.18	+	−	+	−
38	0	39.77	21.81	−	+	+	+	+
39	8	36.46	38.45	24.45	+	−	+	−
40	8	38.56	39.6	27.63	+	−	+	−
41	4	NA	NA	NA	+	−	+	−
42	7	39.51	29	−	−	+	−	+
43	5	38.02	38.94	−	+	−	+	−
44	1	39.09	39.42	−	+	−	+	−
45	0	38.59	−	−	−	−	−	−
46	3	33.22	33.74	23.56	+	+	+	−
47	6	35.44	36.66	29.28	−	−	−	−
48	0	28.87	29.48	27.29	−	−	−	−
49	8	NA	NA	NA	+	−	+	−
50	3	32.45	32.35	23.33	+	+	+	−
51	8	35.93	36.5	25.02	+	−	+	−
52	13	32.37	32.99	27.93	−	−	+	−
53	2	34.33	35.39	23.6	+	+	+	−
54	8	31.4	32.86	23.98	+	+	+	+
55	13	33.08	33.96	23.23	+	−	+	−
56	2	27.46	27.81	22.66	−	−	−	−
57	7	35.89	37.41	23.62	+	−	+	−
58	3	36.36	34.78	25.23	+	−	+	−
59	6	31.12	31.31	23.65	+	−	+	−
60	3	37.08	38.56	26.9	−	−	+	−
61	7	36.77	37.13	23.78	−	−	+	−
62	3	39.33	39.21	23.13	+	−	+	−
63	0	37.14	36.69	22.29	+	−	+	+
64	11	38.46	39.49	22.64	−	−	−	−
65	6	23.5	23.91	21.98	+	+	+	+
66	2	30.41	30.14	24.8	−	−	+	−
67	7	35.46	35.64	24.16	+	−	+	−
68	5	38.36	−	23.94	+	+	+	+
69	11	NA	NA	NA	−	−	−	−
70	2	32.45	32.35	23.33	+	−	+	−
71	6	36.5	36.48	23.72	−	−	−	−
72	6	34.33	35.39	23.6	+	−	+	−
73	5	35.15	35.04	24.01	+	−	+	−
74	8	−	38.18	22.57	+	−	+	−
75	2	−	35.97	31.27	+	−	+	−
76	2	34.05	30.43	27.47	+	−	+	−
77	5	34.21	35.17	24.7	−	−	−	−
78	5	33.65	34.7	−	+	−	+	−
79	8	31.4	32.86	23.98	+	−	+	−
80	7	35.46	35.64	24.16	+	−	+	−
81	2	30.41	30.14	24.8	+	−	+	−
82	2	36.225	−	−	+	−	+	+
83	0	34.12	33.66	24.54			+	
84	5	34.05	32.67	25.99	+	+	+	+
85	6	31.15	28.17	21.45	+	−	+	−
86	NA	NA	NA	NA	+	−	+	−
87	6	39.42	36.2	0	+	+	+	+
88	6	23.12	17.01	24.02	+	−	+	−
89	6	30.93	27.86	21.02	+	−	+	−
90	3	37.29	34.43	28.46	+	−	+	−
91	5	29.17	28.34	25.78	+	−	+	−
92	6	37.65	36.99	23.64	+	+	+	+
93	1	31.79	28.39	21.05	−	−	−	−
94	NA	NA	NA	NA	+	+	+	+
95	7	38.53	34.57	28.3	+	−	+	+
96	5	35.65	33.73	26.61	+	−	+	−
97	1	29.72	28.42	28.57	+	+	+	+
98	1	18.97	15.8	27.28	−	−	−	−
99	7	39.02	37.03	27.57	+	+	+	+
100	5	37.17	33.83	23.91	−	−	−	−
101	5	32.3	30.87	26.87	+	+	+	−
102	1	29.72	28.42	28.57	+	+	+	+
103	5	29.59	28.28	23.01	+	+	−	−
104	1	36.68	35.61	26.01	+	−	+	−
105	5	37.47	25.49	−	+	+	+	+
106	5	37.9	−	26.71	+	−	+	−
107	4	30.98	30.18	26.11	+	−	+	−
108	5	37.27	37.12	25.23	−	−	+	−
109	7	36.61	36	27.48	−	−	+	−
110	8	28.94	27.87	25.49	−	−	−	−
111	8	32.89	31.63	25.83	+	−	+	−
112	8	31.07	29.97	27.39	+	−	+	−
113	8	30.78	29.33	25.18	+	−	+	−
114	8	26.2	24.72	24.75	+	−	+	−
115	8	33.07	32.03	26.19	+	−	+	−
116	8	33.84	32.46	25.6	+	+		−
117	8	33.57	32.47	25.48	+	−	+	−
118	12	33.86	33.05	25.01	+	−	+	−
119	11	26.92	23.55	23.93	+	−	+	−
120	11	36.63	36.09	32.51	+	−	+	−
121	8	34.95	34.44	27.3	+	−	+	+
122	6	NA	NA	NA	+	−	+	
123	11	40	37.75	28.36	+	−	+	+

* CT value unavailable due to samples collected from outside the researchers’ laboratory. ** (−) means undetected CT value.

## Data Availability

Data is contained within the figures and tables of the article and supplementary materials.
